# Violent Behaviours among Adolescents and Young Adults: Association with Psychoactive Substance Use and Parenting Styles

**DOI:** 10.3390/ijerph19073756

**Published:** 2022-03-22

**Authors:** Elisa Benedetti, Emanuela Colasante, Sonia Cerrai, Gilberto Gerra, Leonardo Tadonio, Pietro Pellegrini, Sabrina Molinaro

**Affiliations:** 1 National Research Council, Institute of Clinical Physiology (CNR-IFC), 1 Via Moruzzi, 56124 Pisa, Italy; elisa.benedetti@ifc.cnr.it (E.B.); colasante@ifc.cnr.it (E.C.); sonia.cerrai@ifc.cnr.it (S.C.); 2 Department of Mental Health, AUSL of Parma, Largo Natale Palli 1/A, 43121 Parma, Italy; ggerra@ausl.pr.it (G.G.); ltadonio@ausl.pr.it (L.T.); ppellegrini@ausl.pr.it (P.P.)

**Keywords:** violent behaviour, substance use, parenting, adolescents, ESPAD

## Abstract

This study extends existing research on the relationship between psychoactive substance use among young people and violent behaviour, by evaluating the possible effect of the modification of parenting in a nationally representative sample of 14,685 Italian students drawn from the 2019 wave of the ESPAD Italia survey (51% male; mean age about 17 years). Parental dimensions considered in the study were rule-setting, monitoring, and emotional support, as well as the possible absence of a parent. Relative risk ratios and binary logistic regressions were used to estimate the associations separately for adolescents (15–17) and young adults (18–19). Overall, parental rule-setting, perceived parental monitoring, and emotional support were protective factors for substance use, and the strength of this relationship increased with the frequency of use. Among adolescents, the absence of a parent represented a risk factor. In both age groups, the odds of engaging in violent behaviour was increased among those reporting alcohol intoxication and substance use and the greater the frequency of use, the greater the increase in the odds. As parental monitoring and emotional support decreased, the odds of engaging in violent behaviour increased (except in the case of lower parental support among young adults), while the opposite applies to parental rule-setting. The odds of engaging in violent behaviour were increased among those reporting the absence of a parent only in the adolescent age group. Parental rule-setting was found to have an effect only among adolescents, increasing the odds of violent behaviour among frequent drinkers. Our results might be helpful to signal adolescents who would be more prone to adopt violent behaviour in order to target prevention policies.

## 1. Introduction

Aggressive behaviour and interpersonal violence among adolescents are growing public health issues in Europe [1,2].

Several biological, social, cultural, economic and environmental factors interact to increase young people’s risk of being involved in such behaviours. Among these, existing evidence supports the association between the use of psychoactive substances (such as alcohol or illicit drugs), and violent behaviour [3].

Drinking patterns have been shown to be important predictors of aggressive behaviour in the adult population, with intoxication and heavy episodic drinking in particular increasing the likelihood of aggressive behaviour [4]. Among adolescents, a previous cross-national study based on 13 European countries found that the alcohol-violence association varies across countries with different drinking patterns, and it is stronger in those where intoxication is relatively more prevalent [5]. However, the study did not include Italy, and no evidence is currently available for this country, where alcohol use is common among adolescents, but the prevalence of intoxication is below the European average [6].

Aggressive behaviour was reported to be significantly associated with drinking and drug use by other researchers [1,3,7,8,9,10,11,12,13]. However, the results are still inconsistent, particularly in relation to the different substances and the frequency of use.

In particular, the association between substance use and violent behaviour cannot be interpreted simply as the pharmacological effects of drugs or alcohol on behavioural reactions. Vulnerability conditions characterising adolescence, as well as individual and social factors, may significantly concur with psychoactive substances in increasing risk behaviour.

Among the factors identified to play a major role in child behavioural development, there is the family environment, and, specifically, parents. The relationship with parents, including low parental monitoring and parental support [14], as well as their loss [15] or single parenthood [16], have been identified as risk factors for the adoption of violent behaviour among adolescents. Parents have also been shown to have a critical role in influencing adolescent substance use [17,18,19].

Accordingly, the quality of parenting may influence substance use and violent behaviour trajectories in parallel, even without causal relationships [20,21].

Given the above, the present study had two specific aims: (1) to assess the association between types and patterns of substance use (i.e., alcohol, cannabis and/or other illicit drugs) and the prevalence of violent behaviour; (2) to investigate the association between different dimensions of parenting (perceived rule-setting, monitoring, perceived emotional support and the possible absence of a parent) and the prevalence of violent behaviour, as well as their possible modification effect on the relationship between substance use and violent attitudes.

The 2019 ESPAD Italia survey provides the opportunity to assess these relationships in Italy using a nationally representative sample of adolescents and young adults aged 15–19 years. In particular, as it is part of normal development for young adults aged 18–19 years to become more independent from their parents, they will be considered separately from adolescents aged 15–17.

## 2. Materials and Methods

### 2.1. Data and Design

Data for the present study were drawn from the 2019 wave of the ESPAD Italia cross-sectional survey, conducted every year since 1995 by the Institute of Clinical Physiology of the Italian National Research Council (CNR-IFC). ESPAD Italia is the only nationally representative survey that collects comparable data among 15- to 19-year-old students to monitor trends in drug use and other risk behaviours in Italy. A multistage stratified random sampling is used, with school classes as the last sampling unit. Data are collected through an anonymous questionnaire in the classroom setting through a mixed administration mode (paper-and-pencil or computer-based) under the same conditions as an exam. Participation is voluntary, and pupils can decide not to take part or to withdraw at any time. Concerning the consent procedures, passive parental consent is used. Specific information letters are provided to participating schools, parents/guardians, teachers and pupils illustrating the aims of the project, the survey administration procedure—including all the measures taken to ensure the privacy and anonymity of participants (pupils are requested not to include their name or any other information which could identify them)—as well as the dissemination of results. The results of the study are published only at the aggregate level, no data are presented by single class or school. The study respects the European and national ethics rules and received ethical approval (N. 0027159/2019) from the CNR Research Ethics and Integrity Committee.

The sample consisted of 14,685 students who participated in the 2019 data collection; 51% were male, and the mean age was about 17 years (standard deviation: 1.43). Details of sampling and data collection methodology are reported elsewhere [22].

### 2.2. Dependent Variables

Aggressive/violent behaviours were assessed by asking participants how often in the past 12 months they had found themselves in four different situations: hit one of the teachers; taken part in a physical fight where a group of friends was against another one; seriously hurt someone badly enough they needed a doctor; used any kind of weapon to get something from a person. For each type of situation, response options were “Yes” or “No”. A “Yes” to at least one of the four situations defined a general indicator of violent behaviour.

### 2.3. Independent Variables

#### 2.3.1. Substance Use

Substance use was assessed through questions concerning any use in the past 12 months of the following substances: alcohol (intoxication); cannabis; any psychoactive substance other than cannabis.

##### Alcohol Intoxication and Cannabis

In the case of alcohol intoxication and cannabis use, three patterns of use were identified: “once or twice”; “3–19 times”; “20 times or more” in the past 12 months. These cut-off points were chosen to indicate occasional, non-frequent and frequent use.

##### Any Illicit Psychoactive Substance Other Than Cannabis

In the case of any illicit psychoactive substance other than cannabis, the use of at least one of the following substances, at least “once or twice” in the past 12 months, was considered: synthetic cannabis; cocaine; crack; ecstasy; methamphetamines; amphetamines; heroin; new psychoactive substances (NPS); inhalants; hallucinogens; stimulants; tranquillisers or sedatives without medical prescription.

#### 2.3.2. Perceived Parental Rule-Setting, Monitoring and Support

Parental rule-setting was assessed by the question: “Does (do) your parent(s) set defined rules about what you can do at home/outside home?”.

Perceived parental monitoring was assessed as follows: “Does (do) your parent(s) know where and with whom you are spending your evenings?”.

Parental emotional support was assessed by the question: “Do you feel emotionally supported by your parent(s)?”.

Response options to the three questions were: “almost always”, “often”, “sometimes”, “seldom”, “almost never”. The three variables were considered continuous on a Likert scale (from 1 = “almost always” to 5 = “almost never”).

#### 2.3.3. Absence of a Parent

The absence of a parent was assessed by the following two questions: “How much are you satisfied with the relationship with your mother?” and “How much are you satisfied with the relationship with your father?”. Response options to the two questions were: “very satisfied”, “satisfied”, “neither satisfied nor dissatisfied”, “not much satisfied”, “absolutely not satisfied”, “there is no such a person”. At least one “there is no such a person” response to the two questions was considered an indicator of the absence of a parent.

### 2.4. Statistical Analysis

Firstly, relative risk ratios adjusted for gender were calculated to assess the relationship between parental rule-setting, monitoring, and support as well as the absence of at least one parent and variables referring to substance use.

Binary logistic regression analyses were then performed to estimate the association between these variables and the prevalence of aggressive/violent behaviour in the past 12 months.

Then, in order to assess the possible modification effect of parental rule-setting, monitoring and support, and the absence of a parent on the relationship between substance use and the prevalence of aggressive/violent behaviour, the variables which showed a significant relative risk ratio and a statistically significant association in the logistic regression were interacted with the different patterns of substance use. Coefficients that did not show any statistical significance are not reported in the result tables.

All analyses were conducted separately for adolescents and young adults and were adjusted for gender. Results are reported as adjusted odds ratios (aOR). All statistical tests were two-sided, and a *p*-value ≤ 0.05 was considered statistically significant. All statistical analyses were performed with Stata version 16 (StataCorp. LLC., Lakeway, TX, USA) and SPSS version 26 (IBM Corp., Armonk, NY, USA).

## 3. Results

The sample was quite balanced in terms of gender and age, with the 15–17 year age cohort roughly representing 60% of the sample. As shown in Table 1, the prevalence of engagement in violent behaviour was 18.3% among adolescents aged 15–17 and 15.7% among young adults aged 18–19.

Table 2 shows that both among adolescents and young adults the prevalence of engagement in violent behaviour in the past year was higher among male students, as well as among subjects who consumed psychoactive substances except cannabis. Furthermore, in both age groups, the share of students reporting violent behaviour was higher among students who report higher frequency of intoxication and cannabis use in the past year.

The prevalence of violent behaviour did not vary significantly between the different degrees of parental rule-setting in both groups, while it significantly increased with decreasing perceived parental monitoring. Concerning perceived parental monitoring, in the 15–17 year-old group the highest prevalence of violent behaviour was found among those who answered “Seldom” (41.9%), while in the 18–19 year-old group the highest prevalence was found among those who answered “Almost never” (31.3%).

The same happens between the different degrees of parental emotional support in both groups, whilst the absence of a parent seemed to have a different role in the two age groups: among minors, students who reported the absence of a parent were significantly more likely to have engaged in violent behaviour in the past year; among young adults, no statistically significant differences in the prevalence of violent behaviour were shown between students who reported the absence of a parent and those who did not.

As shown in Table 3, among young adults, the absence of a parent did not show statistically significant associations with having experienced any kind of alcohol intoxication in the past 12 months, while among adolescents it appears to be a risk factor in experiencing alcohol intoxication 20 times or more during the past year. Parental rule-setting, perceived parental monitoring, and emotional support were protective factors for engaging in these risk behaviours, and the strength of this association increased with the frequency of intoxication both among adolescents and young adults, with the only exception of parental rule setting, which had no significant association with the experience of alcohol intoxication 20 times or more among adults.

In terms of cannabis use, for both adolescents and young adults, all parenting-related factors showed a statistically significant association. The protective role played by parental rule-setting, monitoring, and emotional support increased with the frequency of cannabis use, with the only exception of parental rule setting, which had no significant association with the experience of cannabis use once or twice among adults. The absence of a parent was associated with risk of cannabis use and the strength of the association increased as the frequency of use increased for adolescents, whilst among young adults it was a risk factor only for those experiencing cannabis use 20 times or more during the past year.

In the case of the use of any illicit substance other than cannabis or psychoactive drugs without a doctor’s prescription, almost all parenting-related factors also showed statistically significant associations. Parental rule-setting (only among adolescents), monitoring, and emotional support were protective factors and, although with a borderline significance, the absence of a parent was a risk factor.

As shown in Table 4, Table 5, Table 6 and Table 7, binary logistic regressions evidenced relevant differences between adolescents and young adults who reported the use of psychoactive substances and those who did not, related to the engagement in violent behaviour. Results are illustrated by substance(s).

### 3.1. Alcohol Intoxication

As reported in Table 4, the odds of engaging in violent behaviour was higher in those reporting alcohol intoxication, both among adolescents and young adults and the greater the frequency of alcohol intoxication, the greater the increase in the odds. As parental monitoring and emotional support decreased, the odds of engaging in violent behaviour increased (except for parental support among young adults), while the opposite applies to parental rule-setting. Only among adolescents were the odds of engaging in violent behaviour significantly increased among those reporting the absence of a parent.

When testing the possible modification effect of parental rule-setting on the relationship between the different patterns of alcohol intoxication and engagement in violent behaviour, we found a statistically significant influence on the odds only among adolescents.

Table 5 shows that decreasing parental rule-setting increased the odds of violent behaviour among students who reported to have had 20 or more experiences of alcohol intoxication in the past 12 months.

Among students reporting alcohol intoxication 1–19 times in the past 12 months, parental rule-setting did not show any statistically significant influence on the odds of engagement in violent behaviour.

### 3.2. Cannabis Use

The odds of engaging in violent behaviour increased with cannabis use (Table 6) and, as in the case of alcohol intoxication, the odds were higher in both adolescents and young adults who reported a higher frequency of cannabis use in the past year. Parental rule-setting was a risk factor while parental monitoring was a protective factor. Only among adolescents, the odds of reporting a violent behaviour decreased as emotional support from parents increased, while they increased in those reporting the absence of a parent.

When testing the possible modification effect role of perceived parental monitoring, emotional support, and the absence of a parent, the results (not reported here) did not show any overall statistical significance.

### 3.3. Any Illicit Psychoactive Substance Other Than Cannabis

Table 7 shows that the odds of engaging in violent behaviour increased among those reporting the use of any illicit psychoactive substance other than cannabis in the past 12 months, both for adolescents and young adults.

Parental rule-setting was a risk factor while parental monitoring was a protective factor. Only among adolescents, the odds of engaging in violent behaviour decreased as perceived emotional support increased, while they increased among those reporting the absence of a parent.

When testing the possible modification effect function of parental rule-setting, perceived parental monitoring, emotional support, and the absence of a parent on behaviour, the results (not reported here) did not show any overall statistical significance.

## 4. Discussion

Our study contributes to the growing literature investigating the links between psychoactive substance use and aggressive/violent behaviour among young people, by taking into account alcohol, cannabis, and other illicit drugs and different patterns of use. Considering the growing interest in the study of interpersonal violence among adolescents as a public health issue in Europe [1,2], our results based on an Italian nationally representative sample can offer an interesting insight. In order to provide helpful information to identify adolescents who would be more prone to adopt violent behaviour for the targeting of prevention policies, the present study also investigates the role of parenting, a recognised factor playing a crucial role in behavioural issues among adolescents [23,24]. It does so by identifying the dimensions of parenting styles that can contribute to lowering the odds of adopting such behaviour and studying whether they can play a modification effect role in the relationship between violence and substance use.

Our findings confirmed the existence of a strong link between the use of psychoactive substances for non-medical purposes, alcohol intoxication, and violent behaviour among adolescents. Despite the decline observed over the past decade, alcohol use is still common among adolescents and young adults. Our findings confirm that, even in drinking cultures such as the Italian one, where intoxication is relatively less prevalent [6], drinking patterns are associated with violence and, confirming previous findings among adults [4], the frequency of intoxication occasions significantly increases the likelihood of aggressive behaviour, regardless of the considered age range. This is also in agreement with previous studies on high school student samples [5,25,26,27]. The interpretation of this relationship is not easy, considering that alcohol has been found to have a stronger effect on the more violence-prone youth and intoxication effects may reflect social factors rather than the pharmacological effects of alcohol [28]. In fact, adolescents with high rates of violence across relationships have been previously reported to be exposed to increased alcohol misuse [29]. From this perspective, alcohol would be a facilitator, rather than an instigator of aggressive and violent behaviour.

Our results do not support the findings of a recent similar study carried out among 16-year-old students in Romania [21], which did not find cannabis use to be predictive of violent behaviour. However, that study used a dichotomous measure of cannabis use over a lifetime, which could even indicate an experimental use, while our study focuses on more recent use, taking into account different frequencies of use in the past year, from occasional use (1–2 times) to frequent use (20 times or more). We found that the odds of adopting violent behaviour is increased among students who reported higher frequency of cannabis use in the past year, regardless of their age group. This finding is in line with the results from two other studies based on teenage students [30,31], and in particular with the one conducted in the Netherlands [31], which used similar measurements, and found that a significant association between delinquent and aggressive behaviour and cannabis use was present only among those who had used cannabis in the past year and increased with the frequency of use. Our results are also in line with other studies analysed in a recent meta-analysis work, which indicates that cannabis use among young men and women is moderately associated with physical violence [32] and suggests a potential dose-response relationship between frequency of use and physical aggression. The reasons for this association remain unclear since the acute effects of cannabis generally comprise mild euphoria and relaxation [33]. Externalising behaviours such as physical violence have been reported to possibly precede cannabis use rather than the other way around among young people [34]. In the same way, our results confirm recent research indicating other substance use, cocaine [35] and polysubstance [36], in particular, as risk factors for the prevalence of aggressive behaviour in adolescents and young adults. An interesting perspective is that cannabis and other drug use, and particularly frequent use at a young age, are only a part of deviant behaviour patterns involving risk-taking behaviour such as aggressive acts [31]. Furthermore, one possible mechanism is that the use of drugs brings people into contact with the illegal drug market and drug dealers, which in turn might encourage involvement in other risky situations [37]. As in the case of alcohol and cannabis, our cross-sectional study does not allow an investigation of causal relationships and to understand whether the exposure to drugs would have exerted more extensive effects on brain functions with the related behavioural problems, or if pre-existing early problematic experiences might have contributed to vulnerability for both violence and drug use. It is worthy of note, that no substantial differences were detected considering the prevalence of violent behaviour in relation to the use of psychoactive substances between minors and young adults, suggesting a similar vulnerability in these early ages.

On the other hand, our findings provide relevant information on the relationship between perceived parenting practices and substance use as well as violent behaviour, where we find interesting differences between adolescents and young adults. In particular, among adolescents, higher parental rule-setting decreased the risk of lower frequencies of alcohol intoxication and of all patterns of cannabis and any other drug use. Parental rule-setting seemed to have a weaker role among young adults, especially when considering the occasional use of cannabis and other illicit substance consumption. When looking at the relationship with violent behaviour, parental rule-setting assumed an opposite effect, increasing the odds of committing violent acts. Furthermore, only among adolescents, rule-setting also acted as an effect modifier decreasing the odds of violent behaviour among heavy drinkers (alcohol intoxication 20 times or more). The mixed role of this parenting dimension could possibly indicate a subjective perception of rule-setting that depends very much on what rules are being set and how they are enforced. Another possible explanation is that parental rule-setting might be linked to higher strictness and control preventing students from attending environments which ease the approach to substances, whilst in the case of violent behaviour, these might face the oppositional defiance to disagreed and imposed rules typical of young ages. In terms of perceived parental monitoring, lower levels increased the risk of all substance use and related consumption patterns, as well as the odds of engaging in violent behaviour in both the age groups. Perceived parental emotional support showed similar patterns, even though the relationship with the prevalence of violent behaviour lost its statistical significance among young adults. This highlights the protective role of positive family influences, especially among minors. In particular, it suggests that a parenting style combining strictness and warmth, defined as authoritative [38], might be the most effective in preventing risk behaviour, comprising substance use and violent behaviour. This confirms previous studies [39,40], which suggested that this style reduces externalising problems via increased self-esteem, matureness and competence. Differently, following this line, the mixed role of rule-setting in our results might be attributable to the variation in adolescents’ reactions to rule-setting, which highlighted how some responses are constructive and others dysfunctional due to the oppositional defiance to disagreed and imposed rules typical of adolescence and the inhibition of adolescents’ sense of autonomy [39,40,41,42].

The absence of a parent was found to increase the risk of alcohol intoxication (20 times or more) and all patterns of cannabis use among 15–17-year-old students, while it only increased the risk of frequent cannabis use (20 times or more) and had a borderline association with other substance use results in the young adult group. For the adolescent group, the odds of adopting violent behaviour was increased by the absence of a parent in the three models, whilst it was not in the young adult group. This is in line with a recent systematic review [16] revealing that growing up in single-parent families is associated with an elevated risk of problematic behaviour by adolescents and that more research is needed to determine the effects of the different constituting events of single-parent families (divorce/separation or parental decease), which however could not be investigated in our study.

Overall, our results seem to suggest that while a combination of parental rule-setting, effective monitoring, and support could be the best approach to substance use by adolescents, the strictness component might not achieve the intended outcome in relation to violent behaviour, to the point of being counterproductive. As adulthood approaches, parental rule-setting does not seem to protect from experimenting in the use of illicit substances (once or twice) but maintains its role for higher frequencies of use. The absence of one or both of the reference figures in the early stages of development seems, instead, to be confirmed as a risk across all relationships considered, while it is less influential once legal adulthood, which constitutes a turning point towards matureness and responsibility, is reached.

Overall, our results suggest that, particularly among adolescents, ensuring the presence of parents who are able to combine effective monitoring strategies with warmth may be the primary means to prevent overall risk behaviour, including both substance use and aggressive/violent acts.

## 5. Limitations

The limitations of this study include the cross-sectional design that is unable to determine causal relationships and self-reported data, which entail the risk of socially desirable answers. Furthermore, the ESPAD survey is not designed for the in-depth evaluation of parental practices or violent behaviour and our dichotomised approach may have failed to detect relevant differences within parenting practices or violent behaviour. Finally, there might be other factors not investigated in the present study, such as social isolation, which might increase the likelihood of deviant behaviour, including cannabis use and violence, which deserve further research.

## 6. Conclusions

Our findings indicate that the prevalence of violent behaviour among young people is part of a broader risk profile, and that addressing substance use through community- and school-level prevention actions to reduce violence might be a useful strategy. In particular, the association found with substance use might be helpful to identify adolescents who may be more prone to adopt violent behaviour, in order to target policies. This is especially relevant in historical moments when limited public resources are available, as comprehensive approaches simultaneously addressing both behaviours might not only be highly beneficial, but also cost-saving. Furthermore, our study highlighted the relevant role of parents, suggesting that a good approach may include specific actions involving teenagers’ caregivers to improve parental skills and the parent-child relationship.

## Figures and Tables

**Table 1:** Descriptive statistics.

		Adolescents (15–17)		Young Adults (18–19)	
		*n*	%	*n*	%
Gender	Male	4505	50.8	2912	51.1
	Female	4363	49.2	2787	48.9
Alcohol intoxication	Never	6806	78.0	3464	61.5
	1–2 times	1428	16.4	1456	25.9
	3–19 times	457	5.2	618	11.0
	20 times or more	39	0.4	91	1.6
Cannabis use	Never	6897	80.1	3627	65.4
	1–2 times	641	7.4	638	11.5
	3–19 times	674	7.8	730	13.2
	20 times or more	403	4.7	551	9.9
Any psychoactive substance use other than cannabis	No	8112	92.2	5103	90.0
	Yes	684	7.8	568	10.0
Violent behaviour	No	6491	81.7	4351	84.3
	Yes	1451	18.3	807	15.7
Parental rule-setting	(1) Almost always	2205	27.0	963	18.2
	(2) Often	2352	28.8	1374	26.0
	(3) Sometimes	1901	23.2	1349	25.5
	(4) Seldom	893	10.9	811	15.4
	(5) Almost never	828	10.1	788	14.9
	mean	2.5		2.8	
	sd	1.3		1.3	
Perceived parental monitoring	(1) Almost always	5212	63.9	3119	59.2
	(2) Often	1930	23.7	1321	25.1
	(3) Sometimes	597	7.3	482	9.2
	(4) Seldom	216	2.6	165	3.1
	(5) Almost never	201	2.5	184	3.5
	mean	1.6		1.7	
	sd	0.9		1.0	
Parental emotional support	(1) Almost always	4535	55.9	2722	51.7
	(2) Often	2094	25.8	1436	27.3
	(3) Sometimes	939	11.6	644	12.2
	(4) Seldom	302	3.7	253	4.8
	(5) Almost never	246	3.0	207	3.9
	mean	1.7		1.8	
	sd	1.0		1.1	
Absence of a parent	No	8051	96.9	5130	95.5
	Yes	262	3.1	240	4.5

Note: Adolescents *n*. = 8943, young adults *n*. = 5742.

**Table 2:** Differences in prevalence of violent behaviour by demographic data and other independent variables.

		Adolescents (15–17)			Young Adults (18–19)		
		*n*	%	chi2	*n*	%	chi2
				(*p*-Value)			(*p*-Value)
Gender	Male	953	24.6	0.000	558	21.9	0.000
	Female	482	12.0		243	9.4	
Alcohol intoxication	Never	927	15.1	0.000	351	11.2	0.000
	1–2 times	335	27.0		238	18.1	
	3–19 times	131	34.1		160	29.6	
	20 times or more	19	64.5		41	51.3	
Cannabis use	Never	936	15.0	0.000	360	10.9	0.000
	1–2 times	130	23.0		94	16.4	
	3–19 times	182	31.4		159	24.6	
	20 times or more	139	44.5		167	35.6	
Any psychoactive substance use other than cannabis	No	1252	17.3	0.000	648	14.1	0.000
	Yes	182	30.7		150	30.1	
Parental rule-setting	(1) Almost always	382	18.3	0.258	146	15.9	0.512
	(2) Often	383	17.0		197	14.8	
	(3) Sometimes	342	18.8		220	16.9	
	(4) Seldom	154	17.9		126	16.2	
	(5) Almost never	161	20.4		111	14.4	
Perceived parental monitoring	(1) Almost always	677	13.6	0.000	365	12.0	0.000
	(2) Often	407	22.0		212	16.9	
	(3) Sometimes	182	32.2		118	25.8	
	(4) Seldom	87	41.9		44	28.4	
	(5) Almost never	64	34.5		55	31.3	
Parental emotional support	(1) Almost always	689	15.9	0.000	370	14.0	0.000
	(2) Often	366	18.3		204	14.8	
	(3) Sometimes	197	21.9		122	19.9	
	(4) Seldom	89	31.1		56	22.8	
	(5) Almost never	70	30.1		41	21.0	
Absence of a parent	No	1358	17.9	0.000	763	15.6	0.806
	Yes	67	26.8		36	16.2	

**Table 3:** Relative risk ratios (RRR) adjusted for gender.

		Adolescents (15–17)		Young Adults (18–19)	
		aRRR	*p*-Value	aRRR	*p*-Value
Alcohol intoxication (1–2 times) ^a^	Parental rule-setting	1.07	0.003	1.09	0.000
	Perceived parental monitoring	1.38	0.000	1.21	0.000
	Parental emotional support	1.21	0.000	1.13	0.000
	Absence of a parent (Yes vs. No)	1.34	0.050	0.93	0.616
Alcohol intoxication (3–19 times) ^a^	Parental rule-setting	1.08	0.032	1.09	0.006
	Perceived parental monitoring	1.64	0.000	1.44	0.000
	Parental emotional support	1.37	0.000	1.22	0.000
	Absence of a parent (Yes vs. No)	1.26	0.352	1.06	0.751
Alcohol intoxication (20 times or more) ^a^	Parental rule-setting	1.22	0.097	1.06	0.481
	Perceived parental monitoring	1.82	0.000	1.71	0.000
	Parental emotional support	1.66	0.000	1.27	0.007
	Absence of a parent (Yes vs. No)	3.24	0.026	0.00	1.000
Cannabis use (1–2 times) ^b^	Parental rule-setting	1.12	0.000	1.05	0.133
	Perceived parental monitoring	1.31	0.000	1.20	0.000
	Parental emotional support	1.19	0.000	1.13	0.001
	Absence of a parent (Yes vs. No)	1.68	0.009	1.24	0.243
Cannabis use (3–19 times) ^b^	Parental rule-setting	1.19	0.000	1.10	0.001
	Perceived parental monitoring	1.50	0.000	1.39	0.000
	Parental emotional support	1.35	0.000	1.20	0.000
	Absence of a parent (Yes vs. No)	1.98	0.000	1.15	0.457
Cannabis use (20 times or more) ^b^	Parental rule-setting	1.25	0.000	1.23	0.000
	Perceived parental monitoring	1.88	0.000	1.67	0.000
	Parental emotional support	1.67	0.000	1.40	0.000
	Absence of a parent (Yes vs. No)	2.85	0.000	1.71	0.006
Any illicit substance use other than cannabis or psychoactive drugs without a doctor’s prescription (Yes) ^c^	Parental rule-setting	1.08	0.007	1.05	0.143
	Perceived parental monitoring	1.37	0.000	1.25	0.000
	Parental emotional support	1.40	0.000	1.27	0.000
	Absence of a parent (Yes vs. No)	1.43	0.055	1.42	0.049

^a^ Alcohol intoxication (Never) is the reference category; ^b^ cannabis use (Never) is the reference category; ^c^ any illicit substance use other than cannabis or psychoactive drugs without a doctor’s prescription (No) is the reference category.

**Table 4:** Binary logistic regression models between alcohol intoxication in the past 12 months, parenting indicators, and violent behaviour among adolescents and young adults.

	Adolescents (15–17)		Young Adults (18–19)	
	aOR	*p*-Value	aOR	*p*-Value
Alcohol intoxication (1–2 times)	1.96	0.000	1.65	0.000
Alcohol intoxication (3–19 times)	2.60	0.000	3.03	0.000
Alcohol intoxication (20 times or more)	8.65	0.000	6.86	0.000
Parental rule-setting	0.94	0.022	0.92	0.015
Perceived parental monitoring	1.34	0.000	1.26	0.000
Parental emotional support	1.11	0.001	1.06	0.174
Absence of a parent (Yes)	1.65	0.002	1.08	0.698

Note: Adolescents *n*. = 8507, AIC = 0.767; young adults *n*. = 5539, AIC = 0.704.

**Table 5:** Binary logistic regression model between alcohol intoxication, parenting indicators, and violent behaviour in the past 12 months among adolescents: testing the modification effect of parental rule-setting.

	Adolescents (15–17)	
	aOR	*p*-Value
Alcohol intoxication (1–2 times)	1.51	0.020
Alcohol intoxication (3–19 times)	1.68	0.075
Alcohol intoxication (20 times or more)	0.30	0.345
Parental rule-setting	0.90	0.001
Perceived parental monitoring	1.33	0.000
Parental emotional support	1.12	0.001
Absence of a parent (Yes)	1.64	0.003
Alcohol intoxication (1–2 times) * parental rule-setting	1.11	0.096
Alcohol intoxication (3–19 times) * parental rule-setting	1.18	0.090
Alcohol intoxication (20 times) * parental rule-setting	3.33	0.007

* = interaction between alcohol intoxication and parental rule-setting. Note: *n*. = 8507, AIC = 0.766.

**Table 6:** Binary logistic regression models between cannabis use, parenting indicators, and violent behaviour in the past 12 months among adolescents and young adults.

	Adolescents (15–17)		Young Adults (18–19)	
	aOR	*p*-Value	aOR	*p*-Value
Cannabis (1–2 times)	1.57	0.000	1.58	0.000
Cannabis (3–19 times)	2.24	0.000	2.36	0.000
Cannabis (20 times or more)	2.89	0.000	3.26	0.000
Parental rule-setting	0.94	0.027	0.91	0.006
Perceived parental monitoring	1.35	0.000	1.24	0.000
Parental emotional support	1.10	0.004	1.03	0.449
Absence of a parent (Yes)	1.63	0.003	1.02	0.926

Note: Adolescents *n*. = 8394, AIC = 0.765; young adults *n*. = 5468, AIC = 0.707.

**Table 7:** Binary logistic regression models between any psychoactive substance use other than cannabis, parenting indicators, and violent behaviour in the past 12 months among adolescents and young adults.

	Adolescents (15–17)		Young Adults (18–19)	
	aOR	*p*-Value	aOR	*p*-Value
Any illicit substance use other than cannabis or psychoactive drugs without a doctor’s prescription (Yes)	2.24	0.000	2.76	0.000
Parental rule-setting	0.94	0.019	0.93	0.019
Perceived parental monitoring	1.41	0.000	1.32	0.000
Parental emotional support	1.10	0.003	1.03	0.535
Absence of a parent (Yes)	1.71	0.001	0.97	0.883

Note: Adolescents *n*. = 8549, AIC = 0.778; young adults *n*. = 5571, AIC = 0.719.

## Data Availability

Restrictions apply to the availability of these data. Data were obtained from the ESPAD Italia project and are available upon request.
